# Combining experimental and mathematical modeling to reveal mechanisms of macrophage-dependent left ventricular remodeling

**DOI:** 10.1186/1752-0509-5-60

**Published:** 2011-05-05

**Authors:** Yu-Fang Jin, Hai- Chao Han, Jamie Berger, Qiuxia Dai, Merry L Lindsey

**Affiliations:** 1 Department of Electrical and Computer Engineering, University of Texas at San Antonio, San Antonio, USA; 2 Department of Mechanical Engineering, University of Texas at San Antonio, USA; 3 Department of Medicine, University of Texas Health Science Center at San Antonio, San Antonio, USA

## Abstract

**Background:**

Progressive remodeling of the left ventricle (LV) following myocardial infarction (MI) can lead to congestive heart failure, but the underlying initiation factors remain poorly defined. The objective of this study, accordingly, was to determine the key factors and elucidate the regulatory mechanisms of LV remodeling using integrated computational and experimental approaches.

**Results:**

By examining the extracellular matrix (ECM) gene expression and plasma analyte levels in C57/BL6J mice LV post-MI and ECM gene responses to transforming growth factor (TGF-β_1_) in cultured cardiac fibroblasts, we found that key factors in LV remodeling included macrophages, fibroblasts, transforming growth factor-β_1_, matrix metalloproteinase-9 (MMP-9), and specific collagen subtypes. We established a mathematical model to study LV remodeling post-MI by quantifying the dynamic balance between ECM construction and destruction. The mathematical model incorporated the key factors and demonstrated that TGF-β_1 _stimuli and MMP-9 interventions with different strengths and intervention times lead to different LV remodeling outcomes. The predictions of the mathematical model fell within the range of experimental measurements for these interventions, providing validation for the model.

**Conclusions:**

In conclusion, our results demonstrated that the balance between ECM synthesis and degradation, controlled by interactions of specific key factors, determines the LV remodeling outcomes. Our mathematical model, based on the balance between ECM construction and destruction, provides a useful tool for studying the regulatory mechanisms and for predicting LV remodeling outcomes.

## Background

Myocardial infarction (MI) is a leading cause of congestive heart failure (CHF) [1,2]. In response to the MI stimulus, the left ventricle (LV) undergoes structural and functional adaptations that collectively have been termed as LV remodeling [3]. Adverse LV remodeling progresses to CHF in about 25% of post-MI patients, but the mechanisms that drive this progression remain poorly understood. During LV remodeling, both extracellular matrix (ECM) degradation and synthesis increase [4]. When ECM degradation dominates over synthesis, LV rupture can occur. When ECM synthesis dominates over degradation rates, fibrosis can occur. Fibrosis increases myocardial stiffness and further depresses LV function to culminate in CHF [5,6]. Therefore, understanding what regulates the balance between ECM degradation and synthesis post-MI is critical to understand the mechanisms of LV remodeling and may allow us to target specific early diagnostic indicators to better guide treatment protocols.

Previous studies have shown that matrix metalloproteinases (MMPs) regulate ECM degradation and fibroblasts regulate ECM synthesis [7-9]. MMP-9, transforming growth factor-β_1 _(TGF-β_1_), tissue inhibitor of metalloproteinase-1 (TIMP-1), and collagen I levels are significantly elevated from day 1 to day 7 post-MI [4,10,11]. These increases are concomitant with increased infiltration of macrophages and activation of fibroblasts [12]. LV remodeling is a complex process that involves the spatiotemporal interactions among many biological components that remains poorly understood, in part due to the lack of complete sets of experimental data and computational models. Therefore, the objectives of this study were to 1) identify candidate biomarkers of LV remodeling post-MI from ECM gene expression and plasma analyte analyses, and 2) establish a mathematical model that incorporates experimental results to predict LV remodeling outcomes following different interventions. This model would provide a tool to elucidate LV regulatory mechanisms, estimate un-measurable variables, and predict outcomes following multiple therapeutic scenarios.

## Results

### Identifying Key Factors

The key factors were pre-targeted by examining the most significant changes in ECM gene expression in the infarct region at day 7 post-MI, compared to gene expression in the remote non-infarcted region of the same LV and in the LV from control group. In the ECM gene array analysis, total RNA yield was 1.0 ± 0.1, 1.9 ± 0.2, and 2.9 ± 0.3 μg/mg LV tissue for control, remote, and infarct samples, respectively (p < 0.05 for control vs remote and infarct, and for remote vs infarct). Of the 84 genes examined, 51 genes were differentially expressed among control, remote, and infarcted groups (all p < 0.05). The most prevalent pattern of gene expression changes was an increased expression level in the infarct tissue, compared to both control and remote groups. Of the 51 genes, 17 genes showed > 2.5-fold change in the infarct region, and these genes are listed in Table 1. Of the 17 genes with >2.5-fold change, the most significantly over expressed genes are cadherin 3, collagen 1, collagen 2, and collagen 3, osteopontin, periostin, tissue inhibitor of metalloproteinase-1, fibronectin, secreted protein acidic and rich in cysteine (SPARC), and transforming growth factor-β. From this list, collagen I was selected as a key factor, because it is the major collagen in the normal LV, accounting for 90% of cardiac ECM. Osteopontin expression levels increased 206-fold in the infarct region compared to the control group, suggesting strong macrophage activation at day 7 post-MI. TIMP-1 expressions increased 31-fold in the infarct compared to the control group, suggesting strong inhibition of proteolytic activity. Periostin gene expression level was increased 5.5-fold, and TGF-β1 gene expression level was increased 2.6-fold in the infarct sample at day 7, suggesting significant fibroblast functions post-MI [13-16].

**Table 1:** ECM Gene Array (Data are Mean ± SD normalized levels.)

Gene	Control	Remote	Infarct
Cdh3	1.0E-05 ± 9.8E-06	7.4E-05 ± 5.5E-05	2.8E-04 ± 1.3E-04

Col1a1	1.5E-02 ± 2.6E-03	1.9E-01 ± 1.0E-01	8.2E-01 ± 3.4E-01

Col2a1	3.1E-06 ± 6.3E-07	1.1E-05 ± 1.1E-05	2.3E-04 ± 1.8E-04

Col3a1	5.0E-03 ± 1.5E-03	8.0E-02 ± 5.5E-02	2.3E-01 ± 9.7E-02

Col5a1	8.0E-3 ± 2.0E-3	2.3E-2 ± 1.1E-2	4.1E-2 ± 1.6E-2

Ctgf	2.5E-02 ± 4.3E-03	7.6E-02 ± 5.0E-02	1.7E-01 ± 9.1E-02

Fn1	9.0E-03 ± 2.5E-03	4.3E-02 ± 2.7E-02	1.6E-01 ± 6.9E-02

Mmp2	3.5E-03 ± 1.2E-03	1.2E-02 ± 7.9E-03	1.8E-02 ± 7.5E-03

Mmp14	1.9E-03 ± 4.6E-04	6.8E-03 ± 3.2E-03	1.5E-02 ± 6.8E-03

Ncam1	6.2E-04 ± 1.3E-04	1.8E-03 ± 1.3E-03	4.1E-03 ± 1.7E-03

Postn	5.3E-03 ± 1.6E-03	1.5E-01 ± 9.2E-02	2.9E-01 ± 1.2E-01

Sparc	1.3E-02 ± 3.7E-03	7.0E-02 ± 3.7E-02	1.1E-01 ± 4.5E-02

Spp1	1.3E-05 ± 5.7E-06	3.1E-04 ± 4.5E-04	2.6E-03 ± 2.2E-03

Tgfbi	2.0E-03 ± 3.3E-04	3.3E-03 ± 1.2E-03	7.1E-03 ± 3.6E-03

Thbs1	7.2E-03 ± 6.7E-03	1.2E-02 ± 7.6E-03	4.0E-02 ± 1.6E-02

Thbs2	9.0E-03 ± 2.3E-03	1.1E-02 ± 3.6E-03	3.9E-02 ± 1.4E-02

Timp1	2.0E-04 ± 9.4E-05	4.1E-03 ± 3.1E-03	6.1E-03 ± 3.3E-03

Control is unoperated mice; MI is 7d post-MI mice.

We further examined plasma profiles at day 7 post-MI to determine if gene level changes were mirrored in the post-MI plasma. In the plasma profiles, 11 proteins increased in the day 7 post-MI samples (Table 2). Of these, increased MMP-9 and TIMP-1 indicate changes in ECM remodeling. There were several proteins that influence macrophage activation and function, including macrophage inflammatory protein-1 and osteopontin that increased in post-MI plasma. Via analysis of the plasma changes post-MI [4,7-12], we further confirmed key factors involved in macrophage and fibroblast functions, namely collagen I, MMP-9, and TIMP-1.

**Table 2:** Multi-analyte Profiling of Control and 7 day Post-MI Plasma

	Control n = 6	7 d MI n = 7	P value
Clusterin (μg/mL)	330 ± 40	510 ± 200	0.046

Cystatin-C (ng/mL)	360 ± 20	530 ± 100	0.008

Eotaxin (pg/mL)	1500 ± 200	1900 ± 400	0.049

Fibrinogen (mg/mL)	12 ± 2	19 ± 5	0.012

Haptoglobin (μg/mL)	83 ± 30	180 ± 50	0.002

Macrophage Inflammatory Protein-1 gamma (ng/mL)	24 ± 4	33 ± 9	0.042

Matrix Metalloproteinase-9 (ng/mL)	71 ± 9	96 ± 20	0.009

Myeloperoxidase (ng/(mL)	58 ± 10	100 ± 30	0.006

Osteopontin (ng/mL)	250 ± 60	390 ± 100	0.041

Serum Amyloid Protein (μg/mL)	21 ± 3	33 ± 9	0.013

TIMP-1 (ng/mL)	0.8 ± 0.1	3.7 ± 2.0	0.002

In addition, we examined the ECM productions in isolated cardiac fibroblasts stimulated with TGF-β_1_. Fibroblast ECM array analysis showed that TGF-β_1 _stimulation of cardiac fibroblasts up regulated 5 genes and down regulated 7 genes, which are shown in Table 3. Collagen I expression and SPARC expression levels were doubled, and TIMP-1 expression increased 5-fold in response to TGF-β_1 _stimulation at the concentration of 10 ng/mL. These experimental data indicated a primary regulatory effect of TGF-β_1 _on fibroblast ECM production. Interestingly, the 5 up regulated genes are among the 17 ECM genes that were significantly expressed in the LV, indicating that the cardiac fibroblast is likely the major tissue source for these ECM genes (Table 1).

**Table 3:** Fibroblast ECM array in serum free control and 10 ng/ml TGF-β stimulated fibroblasts

	Serum Free	TGF-β	p Value
ECM/Growth Factors			

Col1a1	4.842 ± 1.399	9.614 ± 3.324	0.028

Col5a1	0.301 ± 0.085	0.605 ± 0.230	0.038

Fbln1	0.012 ± 0.005	0.008 ± 0.004	0.032

Sparc	2.709 ± 0.204	4.932 ± 0.379	<0.001

Tgfbi	0.022 ± 0.016	0.013 ± 0.015	0.009

Cell Adhesion Molecules			

Ncam1	0.043 ± 0.016	0.169 ± 0.054	0.011

Pecam1	0.000060 ± 0.000012	0.000022 ± 0.000011	0.015

Sgce	0.187 ± 0.035	0.146 ± 0.018	0.030

Vcam1	0.301 ± 0.136	0.161 ± 0.088	0.031

MMPs/TIMPs			

Mmp7	0.0000075 ± 0.0000011	0.0000056 ± 0.0000004	0.026

Timp1	0.052 ± 0.022	0.272 ± 0.136	0.035

Timp2	0.326 ± 0.109	0.188 ± 0.050	0.027

Data are Avg ± SEM levels (2^-ΔCT^) normalized to 5 housekeeping genes for n = 4 paired fibroblast sets.

We also examined the correlations between LV wall thickness with 6 genes that were over expressed post-MI. The R^2 ^values were 0.76 for collagen 1α1, 0.64 for collagen 2α1, 0.75 for collagen 5α1, 0.60 for periostin, 0.61 for osteopontin, and 0.63 for TGF-β_1_.

**Figure 1:**
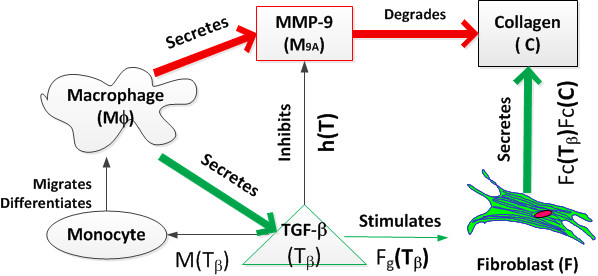
**A working model of post-MI scar formation and remodeling with constructive and destructive modules**. Red arrows represent the destruction pathway and green arrows represent the construction pathway. The regulation scheme includes lines in black, which can be a driving or a triggering stimulus. LV remodeling outcomes are denoted as collagen concentrations in the scar tissue.

In summary, the key factors identified were macrophages, fibroblasts, TGF-β_1_, MMP-9, and collagen. Based on these experimental results, we developed a framework of the interaction loops among the identified key factors (Figure 1).

### Linking Experimental Results to Mathematical Modeling Framework

In the ECM construction pathway, collagen is secreted by fibroblasts. Growth and secretion of fibroblasts are stimulated by TGF-β_1 _(Table 3). In ECM destruction pathway, MMP-9 is a key factor by cleaving collagen. The major source of MMP-9 is the macrophage, which infiltrates into the infarct region post-MI. The majority of macrophages in the LV post-MI are differentiated from peripheral blood monocytes stimulated by chemoattractants including TGF-β_1 _[17]. The major source of TGF-β_1 _is the macrophage. Meanwhile, there are interactions between the ECM construction and destruction pathways: a) MMP-9 regulates ECM construction by activating TGF-β_1 _which stimulates collagen synthesis; b) TGF-β_1 _induces TIMP-1, which inhibits collagen degradation by blocking MMP-9 activity. Linking these key factors with their sources and effects allows us to develop the mathematical model for quantitative analysis.

### Mathematical Modeling

We established a set of nonlinear differential equations to model the temporal interactions among the key factors identified by our experimental results. The model incorporated the following variables: macrophage cell density (M_Φ_, cells/mm^3^), fibroblast cell density (F, cells/mm^3^), collagen concentration (C, μg/μL), TGF-β_1 _concentration (T_β_, pg/μL), and activated MMP-9 concentration (M_9A_, pg/μL). Rates of cell number change were determined by the summation of constructive effects (migration rate or proliferation rate) and destructive effects (death rate or removal rate). Rates of chemical factors (TGF-β_1_, MMP-9, collagen, etc) change were determined by the net difference between the synthesis rate and degradation rate.

Four assumptions were used based on experimental results: 1) All monocytes that migrate to the infarct region are differentiated to macrophages [18]; all activated macrophages are differentiated from peripheral blood monocytes since previous studies have shown that <5% of macrophages undergo mitotic division [19]; 2) The major source of fibroblasts is the resident cell and the contribution of circulating fibroblasts is ignored [20]; 3) The majority (80%) of TGF-β_1 _secreted at the injured site becomes activated [21,22]; 4) ECM proteins in the infarct region are secreted by local cells. Our experimental data showed that ECM gene expression was higher in the infarct region than the expression in the remote region, and ECM gene levels in the remote region were higher than that in the LV of controls, indicating a local source of ECM production.

Accordingly, the scar formation post-MI was modeled by the following set of differential equations(1)(2)(3)(4)(5a)(5b)(6)(7)

The parameters used in these equations with their biological meanings, experimental values, units, and references were listed in Table 4. The interaction functions M(T_β_), *F*_*g*_(*T*_*β*_), and Fc(T_β_), were established based on *in vivo*, and *in vitro *experiments [17,23-26]. We employed the function Fc(C) in a form given by Waugh and colleagues [27,28]. The forms of these functions we took in this study were described in equation 8.(8)

**Table 4:** Pre-determined parameters in the mathematical model

Symbol	Biological meaning	Value	Units	Ref
d_MΦ_	Macrophage removal rate**(eqn 1)	0.6	day^-1^	[29]

ρ_MΦ_	maximal macrophage density (eqn 2)	2500	cells/mm^3^	[28]

ρ_F_	maximal fibroblast density(eqn 2)	1250	cells/mm^3^	[28]

ρ_C_	maximal collagen density (eqn 2)	3300	μg/mm^3^	[28]

k_F_	Fibroblast growth rate* (eqn 3)	0.924	day^-1^	[62]

d_F_	Fibroblast apoptosis rate (eqn 3)	0.12	day^-1^	[33]

k_MΦT_	Macrophage TGF-β production rate (eqn 4)	0.07	pg/cell/day	[34]

k_FT_	Fibroblast TGF-β production rate (eqn 4)	0.004	pg/cell/day	[35]

d_Tβ_	TGF-β degradation rate^¥^(eqn 4)	15	day^-1^	[36]

K_MφM9_	Macrophage secretion MMP9 rate (eqn 5)	3	pg/cell/day	estimated

d_M9_	MMP-9 degradation rate(eqn 5)	0.875	day^-1^	[63-65]

k_on_	Kinetic reaction speed (eqn 5)	3 × 10^-4^	1/(μg/mm^3^)s^-1^	[42,66,67]

k_off_	Kinetic reaction speed (eqn 5)	4 × 10^-4^	s^-1^	[42,66,67]

k_onc_	Kinetic reaction speed (eqn 6)	0.004	s^-1^	[42,66,67]

k_FC_	Fibroblast collagen production rate (eqn 6)	20	μg/cell/day	[41]

*The growth rate of cells was determined by the population doubling time (T_2_) via equation k = ln 2/T_2 _[68].

** Since macrophages emigrate from the scar tissue to lymph node system instead of dying locally in the scar tissue, the removal rate of macrophage, d_MA_, was used in our model.

¥ The decaying rate of chemical factors was calculated from their half-life (T_1/2_) via the equation d = ln 2/T_1/2 _[68].

**Figure 2:**
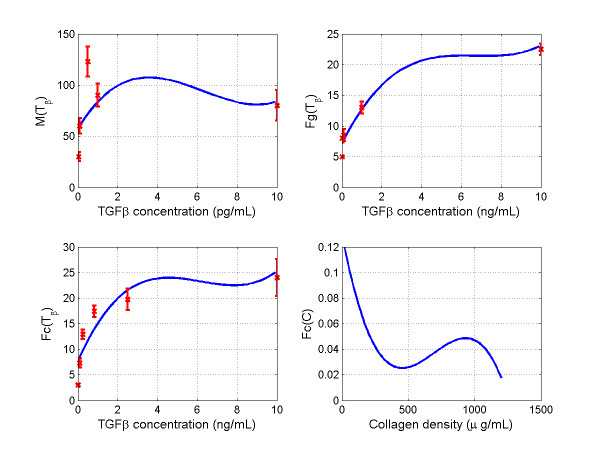
**Macrophage migration rate M(T_β_) **[17], **fibroblast growth rate Fg(T_β_**) [17], **and fibroblast secretion rate Fc(T_β_**) [23,41]**plotted as functions of TGF-β_1 _concentration**. The original experimental data (x, Mean ± SD) are shown for comparison. The function Fc(C) was provided by Waugh et al [27,28].

Plot of these constructed functions and the available experimental data were shown in Figure 2.

Equation 1 determines the rate of macrophage (M_Φ_) infiltration. The function M(T_β_) describes the migration rate of macrophages to the scar tissue [17]. Since we assumed that all monocytes differentiated into macrophages (assumption 1), M(T_β_) also represents the migration of monocytes stimulated by TGF-β_1_. Parameter d_MΦ _denotes the emigration rate of macrophages [29], since macrophages do not die locally in the scar tissue but emigrate to the lymph node system for disposal.

**Figure 3:**
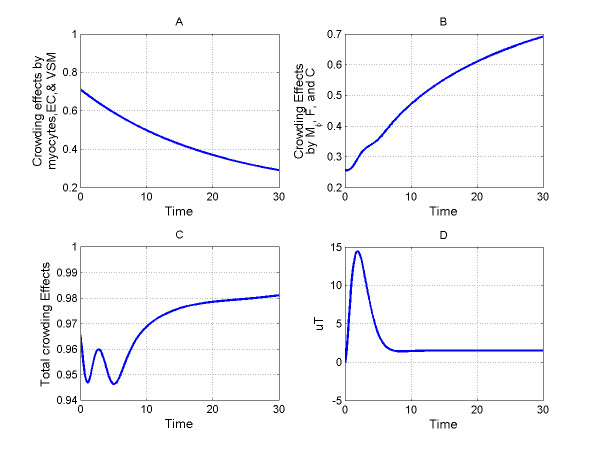
**Plots of crowding effects (equation 2) and TGF-β**_**1 **_**activation function ***u*_*T *_**(equation 4)**. A: Crowding effects (*k*_*mem *_(*t*) in equation 2) contributed by myocytes, endothelial cells, and vascular smooth muscle cells. B: Crowding effects contributed by macrophages, fibroblasts, and collagen in the scar tissue. C: Total crowding effects imposed by the environment. D: TGF-β_1 _activation function, *u*_*T *_according to small scar size. Activation peak time is at day 2 post-MI with amplitude at 15 pg/μL/day.

Equation 2 determines the crowding effect of myocytes, endothelial cells, vascular smooth muscles cells, fibroblasts, macrophages, and collagen in the myocardium, which are affected by total environment density [30,31]. Parameter  denotes the crowding effects contributed by myocytes, endothelial cells, and vascular smooth muscle cells. The parameter 0.17 represents the total percentage of endothelial and smooth muscle cells that account for 7% and 10% of total cell numbers in normal mouse myocardium, respectively [32]. Parameter 0.56 represents the percentage of myocytes (56%) in normal mouse myocardium [32]. Parameter d_mc _= 0.05 represents the rate of myocyte cell death since ischemic myocytes undergo necrosis in the infarct region post-MI. The crowding effect of macrophages, fibroblasts, and collagen was considered by calculating the normalized density with respect to their maximum density in scar tissue [28]. Temporal profiles of total crowding effects, crowding effects contributed by macrophages, fibroblasts, and collagen, and the parameter *k*_*mem *_(*t*) were shown in Figure 3.

Equation 3 determines the rate of fibroblast (F) density changes based on the assumption that majority of fibroblasts come from the proliferation of resident cells (assumption 2). The function *F*_g_(*T*_*β*_) denotes the stimulating effects of TGF-β_1 _on the growth rate of fibroblasts [17]. Parameter d_F _represents the apoptosis rate of the fibroblast [33].

Equation 4 determines the rate of TGF-β_1 _concentration change, wherein k_FT _denotes the TGF-β_1 _secretion rate of fibroblasts [34] and k_MΦT _denotes the TGF-β_1 _secretion rate of macrophages [35] since a major source of TGF-β_1 _in the scar tissue is the activated macrophage. Parameter d_Tβ _represents the degradation rate of TGF-β_1_, which can be calculated from the half life data [36].

TGF-β_1 _gene levels demonstrated temporal progression at the early stage post-MI. Gene expression profile of TGF-β_1 _increased post-MI, peaked at day 2, and returned to normal levels after day 7 in mice post-MI [37]. Since majority of TGF-β_1 _secreted in the infarct is activated (Assumption 3), gene expression profile can be used as an activation pattern of TGF-β_1_. The function, *ut *denotes temporal profile of TGF-β_1 _activation post-MI [38-40] and works as the input in the simulation. It's worth to mention that TGF-β1 activation levels were different with respect to different scar size. A temporal profile of *ut *representing small scar was shown in Figure 3.

Equation 5 determines the rate of activated MMP-9 concentration change. Proteolytic collagen degradation with activated MMP-9 is described in equation 5a, where M_9A_, C, CM_9_, and CID denote activated MMP-9, collagen, binding of MMP-9 and collagen, and degraded collagen peptide concentration, respectively. MMP-9 is inhibited primarily by TIMP-1, and TIMP-1 is induced by TGF-β_1_. Thus, we established an inhibition function h(T) = 1/(1+T_β_/T_βN_) with T_βN _= 6.0 pg/μL to represent the inhibition effect.

Equation 6 determines the rate of collagen concentration changes. Collagen secretion rate by fibroblasts was denoted by parameter*K*_*FC*_. Meanwhile, the function, *F*_*c*_(*T*_*β*_), characterizes effects of TGF-β_1 _on collagen secretion rate by fibroblasts [23,41]. Function, *F*_*c*_(*c*), denotes the effect of collagen density on fibroblast secretion rate [27,28].

Equation 7 determines the concentration change of CM_9_, based on the theoretical model for collagen degradation by MMPs proposed by Popel's group [42,43].

### Computational simulations

Computational simulations of scar formation (collagen deposition) were carried out by solving the nonlinear differential equations with MATLAB. Initial conditions of the fibroblast and macrophage densities were chosen as F(0) = 20 cells/mm^3 ^and M_Φ_(0) = 5 cells/mm^3^. Accordingly, T_β_(0) = 0.21 pg/μL, M_9A_(0) = 7.1 pg/μL, C(0) = 839.5 μg/μL, CM_9_(0) = 447.6 μg/μL were calculated by the equilibriums of equations 1-7 for normal LV. All the simulations shown in this study used the same initial conditions. The initial conditions were chosen based on measurements in the normal LV for both the control and MI groups (MI induced at day 0). The simulations covered the LV remodeling process from day 0 to day 30 post-MI.

### Model validation

**Figure 4:**
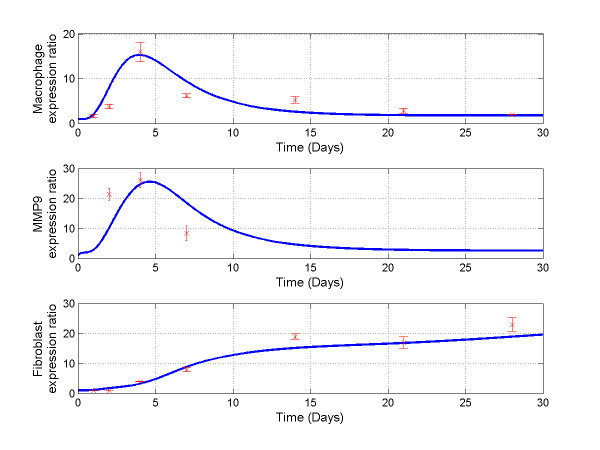
**The relative ratio changes of fibroblast density, macrophage density, and MMP-9 concentrations**. Computational results were normalized to initial conditions and are shown in solid lines. Previously published experimental results were normalized to the corresponding measurements in control group and are shown as x (Mean ± SD). All experiments were carried out in mice with MI induced by coronary artery ligation. The fibroblast and macrophage densities were collected from C57BL/6J mice [12]. MMP-9 profile was collected from 129SV mice [45,46].

To validate our mathematical model, we compared our simulation results to experimental data from our lab or reported in the literature [9,12,44]. We normalized macrophage and fibroblast cell densities and MMP-9 concentrations to the corresponding measurements in the normal LV and plot the experimental data (Mean ± SD) in Figure 4. Our computational simulation results were also normalized to the corresponding initial conditions. This normalization will give us the relative fold changes of macrophage and fibroblast cell densities and MMP-9 concentration. The simulation results showed similar progression trend of cell densities and MMP-9 concentration profiles, peak values, and stable values as the experimental results (Figure 4).

**Figure 5:**
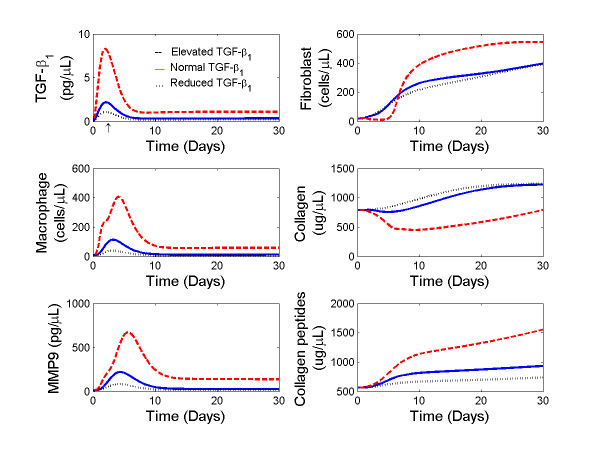
**Temporal profiles of TGF-β**_**1**_**, macrophages, MMP-9, fibroblasts, collagen, and collagen peptides from days 0 to 30 post-MI in response to low (**····**), median (blue line), and elevated (red dash) TGF-β**_**1 **_**stimuli**. The initial conditions were set to F(0) = 20 cells/mm^3 ^and M_Φ_(0) = 5 cells/mm^3^, T_β_(0) = 0.21 pg/μL, M_9A_(0) = 7.1 pg/μL, C(0) = 839.5 μg/μL, CM_9_(0) = 447.6 μg/μL, according to measurements in normal myocardium. The symbol ↑ indicates the peak time of TGF-β_1 _stimulus.

In addition, our simulations correctly predicted MMP-9 responses to three TGF-β_1 _stimuli corresponding to reduced, normal, and elevated post-MI activation strength. Others have reported an early increase of MMP-9 levels of 78 ± 19 pg/μL for small infarcts and 195 ± 63 pg/μL for large infarcts [45,46]. These experimental results agreed with the MMP-9 predictions by our mathematical model, which peak at 80 pg/μL for small infarcts, and 220 pg/μL for large infarcts, respectively. MMP-9 profiles were shown in Figure 5 and details of the simulation setup for Figure 5 were explained as follows.

#### Effects of TGF-β_1 _levels

We employed activation of TGF-β_1_, *u*_*T *_in equation 4, at reduced, normal, and elevated post-MI strength. The activation function peaked at 15, 30, and 60 pg/μL/day, according to active expression levels observed in small, median, and very large infarcts, respectively. Temporal profiles of cell densities of macrophages, fibroblasts, concentrations of MMP-9, collagen, and TGF-β_1 _responses to the stimuli *u*_*T *_are shown in Figure 5. For the TGF-β_1 _stimulus level setting at 30 pg/μL/day (a normal post-MI level in mice), TGF-β_1 _peaked after day 2 post-MI, macrophage density peaked at day 3, MMP-9 concentration peaked at day 4, and all were returned to normal levels at 30 days post-MI. In contrast, fibroblast density and collagen density continued to increase beginning at day 4, reached a stable value after day 20 post-MI, and then remained at a higher equilibrium level at day 30 post-MI (blue solid line in Figure 5). This computational predictions agreed with the experimental observations on the peak time of macrophages [9,12], and progression trend of MMP-9 [10] and collagen for stable LV remodeling [47].

In the case of reduced TGF-β_1 _levels (with an activation peak at 15 pg/μL/day in Figure 3), less monocytes were attracted to the infarct. Thereby, less macrophages and MMP-9 appeared at the early stage. The reduced MMP-9 levels slowed down collagen degradation, leading to a higher collagen concentration (black dotted lines in Figure 5. This simulation agreed with the temporal profiles of macrophage and fibroblast density [9,12], and relative low expression levels of MMP-9 for moderate size of infarcts compared to large infarcts [46,48].

When the TGF-β_1 _stimulus strength was elevated to a level 2-fold higher than normally seen post-MI in mice, more macrophages infiltrated to the infracted region in the early days (Figure 5 red dash lines). Elevated macrophage infiltration led to high levels of crowding effect and high concentrations of MMP-9 for collagen degradation. Therefore, fibroblast growth was inhibited earlier due to the crowding effect, which led to less collagen secretion and a net negative collagen deposition. These results indicated an increased early susceptibility to LV rupture between day 2 to 6 post-MI and prolonged LV remodeling for those mice that did not rupture. Though we know that LV ruptures frequently occurred at day 2-4 post-MI in a normal remodeling process [49], this simulation has not been examined experimentally and raises some interesting hypotheses.

#### Effects of MMP-9 interventions on ECM destruction

**Figure 6:**
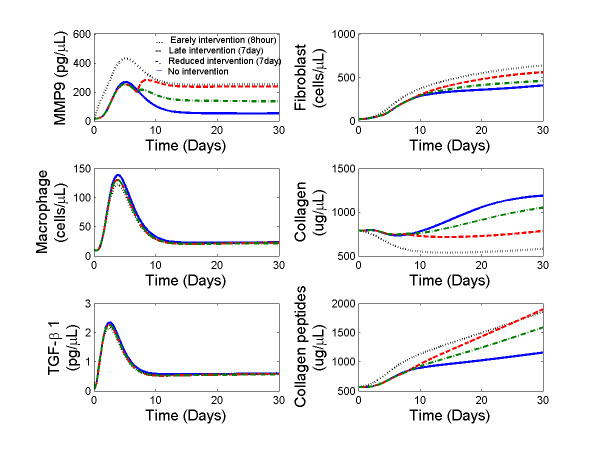
**Temporal profiles of MMP-9, macrophages, TGF-β**_**1**_**, fibroblasts, collagen, and collagen peptides from days 0 to 30 post-MI at different MMP-9 interventions**. The profiles include progressive changes in response to MMP-9 intervention at day 1 (····) and day 7 (red dash), as well as reduced MMP-9 intervention strength at day 7 post-MI (green dot-dash), and no intervention (blue line). The initial conditions were set to F(0) = 20 cells/mm^3 ^and M_Φ_(0) = 5 cells/mm^3^, T_β_(0) = 0.21 pg/μL, M_9A_(0) = 7.1 pg/μL, C(0) = 839.5 μg/μL, CM_9_(0) = 447.6 μg/μL according to the measurements in normal myocardium.

With the validated parameter settings, we also used the mathematical model to predict the effects of MMP-9 interventions at different strengths and intervention times. Specifically, we simulated the LV remodeling responses to three different MMP-9 interventions post-MI: 1) elevation of MMP-9 level (200 pg/μL) beginning at 8 hours post-MI to mimic the earlier increase of MMP-9 levels seen with reperfusion, 2) elevation of MMP-9 levels (200 pg/μL) beginning at 7 days post-MI to mimic a prolonged macrophage infiltration, and 3) reduced elevation of MMP-9 levels (100 pg/μL) beginning at 7 day to mimic therapeutic targeting of MMP-9. The LV remodeling responses were shown in Figure 6. Early MMP-9 intervention (scenario 1) significantly decreased collagen density at day 30 post-MI, consistent with the beneficial effects seen with reperfusion. Elevated MMP-9 levels (scenarios 1, 2, and 3) led to reduced collagen density post-MI, compared to collagen levels seen in LV remodeling outcomes without MMP-9 intervention, when MMP-9 levels would be starting to fall by day 7. Compared to scenario 2, reducing MMP-9 intervention levels at day 7 post-MI (scenario 3) had less effect on collagen degradation, suggesting that the primary effects of MMP-9 might occur before day 7.

## Discussion

This study is the first investigation to integrate mathematical modeling with ECM and fibroblast gene array data and plasma analytes to predict ECM remodeling post-MI. We have integrated *in vivo, in vitro*, and *in silico *approaches to dissect the complicated interactions among multiple regulatory factors in LV remodeling. As such, this study provides a promising modeling approach for elucidating the complicated LV remodeling process. The most important findings of this study are highlighted as follows. 1) The balance between ECM construction and destruction kinetics is likely the key determinant of scar formation, and interventions to either the construction or destruction side lead to different remodeling outcomes. 2) Dynamic interactions among key factors in LV remodeling determine LV remodeling outcomes post-MI. Altering initial conditions, intervention strengths, or intervention times have significant effects on LV remodeling outcomes, and these effects could be simulated accurately by our model. 3) Collagen, MMP-9, TGF-β_1_, and TIMP-1 are critical biomarker candidates of LV remodeling outcomes.

Our experimental results on microarray and plasma data provided the foundations to build our computational framework. We examined 84 ECM genes and chose the 17 genes that were most highly over-expressed in the infarct region compared to both control and non-infarcted groups (>2.5-fold over-expression). The expression levels of several of these factors were further verified by our plasma data at protein level. One interesting finding was that SPP1 (osteopontin) gene expression levels increased 206-fold in the infarct region compared to the control group at day 7 post-MI, suggesting strong macrophage activation. The plasma protein levels of osteopontin increased from 250 ± 60 ng/mL in controls to 390 ± 100 ng/mL in post-MI samples, which adds support for the critical role of macrophages in our mathematical model. Therefore, the primary selection of the most highly changed genes allowed us to focus on the most significant factors at gene level and predict the possible interactions at protein level and cellular level.

An interesting observation was that MMP-9 mRNA levels did not increase in our gene array analysis, but MMP-9 protein levels increased in the plasma data. It is well known that MMP-9 protein levels and activation were increased at day 7 post-MI. This conflicting phenomena is caused by pre-formed MMP-9 proteins stored in leukocytes, which do not rely on increased gene expression to induce MMP-9 secretion and activation [50]. In addition, the shift from a normal ventricle composed of cardiac myocytes, endothelial cells, and smooth muscle cells to an infarcted ventricle comprised primarily of cardiac fibroblasts and inflammatory cells could result in a quantitative no gain in gene expression level but qualitative increase in MMP-9 function due to increased activation and increased substrate availability. Karl Weber's group showed that MMP-9 protein levels increased early post-MI, but the mRNA levels were not elevated [51]. We have also shown that MMP-9 activation increases upon reperfusion in a dog model of cardiac ischemia/reperfusion [7]. These results support our observations in this study and highlight the fact that MMP-9 is primarily regulated at the post-translational level. Furthermore, the fact that MMP-9 gene levels do not change at day 7 post-MI also suggest that MMP-9 should be evaluated at the protein level.

There is limited data available to construct suitable functions of *M*(*T*_*β*_), *F*_*g*_(*T*_*β*_), *F*_*c*_(*T*_*β*_), and *F*_*c*_(*c*). Wahl et al presented a set of *in vitro *data on monocyte chemotaxis induced by TGF-β with concentration ranges from 0.05 pg/mL to 10 pg/mL [17]. Their experiments also provided production of fibroblast growth activity induced with TGF-β concentration range from 10 pg/mL to 10 ng/mL. These data were used in this study to construct the functions of *M *(*T*_*β*_) and *F*_*g*_(*T*_*β*_). Roberts et al have presented a set of data on *in vitro *collagen formation stimulated by TGF-β with the concentration range from 0.0825 pg/mL to 2.5 pg/mL [23]. Ignotz and Massague have examined the effects of TGF-β on collagenases in chick embryo fibroblasts stimulated by TGF-β at a concentration range of 1.25 ng/mL to 25 ng/mL [41]. We have done experiments to quantify the ECM production of cardiac fibroblast stimulated with TGF-β (10 ng/mL). These experimental data were used to build and justify the function, *F*_*c*_(*T*_*β*_). Loftis and colleagues have studied effects of collagen density on cardiac fibroblast behavior and showed elevated fibroblast activities stimulated with higher collagen concentrations (750 μg/mL - 1250 μg/mL) [52]. Wu and colleagues showed that propeptides at lower levels caused about 80% decrease in collagen synthesis compared to control [53]. In this study, we used an *in silico *function *F*_*c*_(*c*) provided by Waugh and colleagues [27,28]. These experiments were done by different groups with different conditions and the *in vitro *experiments might not reflect the *in vivo *interactions. In addition, we established these functions using polynomial interpolation. It's likely that these functions are not the optimal forms to describe the *in vivo *interactions; however, they provide us a baseline for further study.

Our computational results demonstrated that altering the strength of TGF-β_1 _altered LV remodeling outcomes. Elevated TGF-β_1 _levels at the early stage (day 3 post-MI) led to elevated macrophage density and MMP-9 levels, decreased fibroblast secretion of collagen and collagen deposition, and thereby, prolonged the progression of remodeling. Wetzler and colleagues have shown that the prolonged persistence of macrophages at the late phase (after day 7 post-MI) impairs the wound healing process [54]. It has also been shown that elevated TGF-β_1 _levels delays wound healing post-MI [55,56]. Our simulation results are in agreement with these previously published studies.

Simulations of different TGF-β_1 _strengths also shed light on the regulation scheme of ECM construction and destruction. For ECM construction regulation, active TGF-β_1 _stimulated fibroblast proliferation and collagen secretion, which increased the crowding effect. The increased crowding coefficient ramped down fibroblast proliferation and TGF-β_1 _secretion (negative feedback), which slowed down the stimulus for monocytes to migrate into the infarct region. The decrease of monocytes number led to less macrophage infiltration, which then reduced the crowding coefficient. Meanwhile, reduced macrophages lead to less TGF-β_1 _secretion by macrophage, which further slowed down collagen synthesis. For collagen destruction regulation, TGF-β_1 _induced macrophage infiltration, which lead to elevated MMP-9 secretion, elevated collagen degradation, and thereby reduced crowding coefficients. Smaller crowding coefficients lead to elevated fibroblast proliferation and collagen secretion. Notably, there are two types of negative feedback schemes in the mathematical model: degradation (apoptosis or emigration) rates associated with proteins (cells) and the crowding effects. Degradation rates are constants and determine how fast the proteins (cells) can respond to stimuli. Crowding effects are time varying impacts imposed by the environment. Through these regulation schemes, a dynamic balance of collagen construction and destruction can be maintained to generate a stable scar. Furthermore, profiles of crowding effects elucidated the transition from the normal LV to scar tissue with respect to cell types and collagen concentrations (Figure 3).

It is worth mentioning that there exist biological negative feedbacks in our mathematical model. For macrophage density regulation, there was a positive feedback loop containing macrophage and TGF-β_1_: TGF-β_1 _stimulated monocytes migration, leading to macrophage infiltration; macrophages secreted TGF-β_1 _which might attract more macrophages to the infarct site. We observed elevated macrophage density and MMP-9 concentrations corresponding to increased TGF-β_1 _levels (Figure 5). This positive feedback loop was inhibited by emigration of macrophages and degradation of TGF-β_1_. In addition, Wahl et al pointed out that the monocyte chemotactic activity increased in response to low concentrations of TGF-β_1 _stimuli, while the chemotactic activity decreased in response to higher concentrations of TGF-β_1 _[17]. Therefore, as TGF-β_1 _levels continuously increased, infiltration speed of macrophages decreased as shown in figure 2 (M(T_β_)), suggesting a secondary biological inhibition scheme of the TGF-β_1 _-- macrophage positive feedback loop.

There are a few limitations of the mathematical model that resulted to a large degree from the model assumptions. More research is needed to address these limitations and further enhance the models. First, our model calls for accurate determination of MMP-9 activation and inhibition functions. Complete time-course measurements of TIMP-1 and the other three TIMPs (for MMP-9 inhibition) and MMP-3 (for MMP-9 activation) would provide additional details on the regulators of MMP-9 function. Second, the large differences between simulation results and experimental measurement of MMP-9 concentrations before day 4 post-MI in Figure 4 is likely due to the effect of neutrophils, an early source of MMP-9, indicating the need to investigate the role of neutrophils in the early stage (days 1-5) post-MI [7]. This inconsistency of the simulated MMP-9 concentrations and measurements from experimental results is one of the approaches for us to employ more biomarkers and key factors in model development. Third, Interleukin IL-1, IL-6, IL-10, and tumor necrosis factor-α profiles need to be included to better study macrophage activation phenotypes [57]. Fourth, the effects of other chemoattractants on monocyte migration on the LV remodeling need further investigation. The predicted fibroblast density at days 14-30 post-MI was lower than experimental measurements. This might be caused by assumption 2, that the increase of fibroblast numbers post-MI was contributed solely by proliferation of local cells. We will investigate the migration rates of fibroblasts in the future to refine our mathematical model. Regardless of the above limitations, we were able to use our model to compute the progression of macrophages, fibroblasts, and collagen density, MMP-9 and TGF-β_1 _concentrations post-MI.

This systems biology study for LV remodeling can be expanded to include proteomics and cardiac functions in future studies. We employed plasma data in this study since plasma proteins reflect the process of LV remodeling and plasma data are more directly translatable to the clinic. However, we are well aware that measuring tissue protein levels will provide a more direct evaluation of LV remodeling. Further investigation on the ECM proteomics in cardiac samples has been planned in our future research to establish a more complete mathematical model for LV remodeling. Though it is beyond the scope of the current paper, we have previously reported some data on cardiac function [58-60] and our future study will integrate cardiac function into the mathematical model.

The computational model for post-MI LV remodeling developed here illustrated the dynamic interactions among critical factors in LV remodeling. This is the first mathematical model focusing on the protein and cellular interactions post-MI. Thus, this model provides a strong foundation for future studies to build a more comprehensive model that takes into account a more complete set of parameters. The model also provides a tool to guide experimental designs by identifying candidate factors to intervene, the proper intervention time, and doses for effective interventions to achieve the most beneficial outcomes. As an example, we have shown the effects of MMP-9 intervention time and doses on LV remodeling outcomes in this study. Though this model was established based on *in vitro *data and *in vivo *data from mice, the modeling approach can be used to develop models for other scenarios such as the LV remodeling of human MI and LV remodeling under reperfusion conditions.

## Conclusions

In conclusion, we developed a set of differential equations to quantitatively model the dynamic interactions and temporal changes of the key components identified from our experimental results. Predictions of the mathematical model fell well within experimental measurements, particularly with regard to macrophage infiltration and matrix remodeling. This mathematical model provides a powerful tool to better understand how the dynamic balance between ECM construction and ECM destruction influences LV remodeling outcomes.

## Methods

### Mice

All animal procedures were conducted in accordance with the Guide for the Care and Use of Laboratory Animals (National Research Council, 1996) and were approved by the Institutional Animal Care and Use Committee at the University of Texas Health Science Center at San Antonio. Male C57BL/6J wild type adult mice (n = 13) at age 8.0 ± 0.5 months were used. One group (n = 6) served as unoperated controls, while the other group (n = 7) underwent coronary artery ligation for 7 days as described previously [9] and were sacrificed at day 7 post-myocardial infarction.

### In Vivo Procedures

Blood was collected from the jugular vein and placed in a heparinized tube for plasma collection. Tissue was collected for the gene array analysis as described previously[9].

### Microarray and Plasma Analysis

Total RNA was isolated using the TRIzol plus Total RNA purification kit (Invitrogen). The RT^2 ^qPCR Primer Array for Extracellular Matrix and Adhesion Molecules (SuperArray catalog APMM-013A) was used for the gene array. Results were analyzed based on the ΔΔCt method with normalization of raw data to the GAPDH housekeeper gene. Data are presented as average 2^-ΔCT ^levels.

For the fibroblast ECM microarray analysis, cardiac fibroblasts were isolated from adult C57BL/6J mice and stimulated with or without 10 ng/ml TGF-β_1 _for 24 hours[61]. Plasma samples (80 μL) were analyzed for 67 antigens using the quantitative immunoassay panel for mice provided by Rules Based Medicine in Austin, TX (http://www.rulesbasedmedicine.com).

### Statistical Analysis

Control, remote, and infarct LV groups were analyzed by ANOVA, with the Bonferroni post hoc test. Control and MI plasma was analyzed by Students t-test. Unstimulated and TGF-β_1 _stimulated fibroblast groups were analyzed by paired t-test. A p < 0.05 was considered statistically significant.

## Authors' contributions

YFJ, HCH, and MLL designed the research; JB, and QD performed animal experiments. YFJ performed the computational experiments. YFJ, HCH, and MLL analyzed the results and wrote the manuscript. All authors have read and approved the final manuscript.
